# Waste Glass in Cement and Geopolymer Concretes: A Review on Durability and Challenges

**DOI:** 10.3390/polym13132071

**Published:** 2021-06-24

**Authors:** Ayesha Siddika, Ailar Hajimohammadi, Md. Abdullah Al Mamun, Rayed Alyousef, Wahid Ferdous

**Affiliations:** 1 School of Civil and Environmental Engineering, University of New South Wales (UNSW Sydney), Sydney, NSW 2052, Australia; a.siddika@unsw.edu.au; 2 Department of Civil Engineering, Rajshahi University of Engineering & Technology, Rajshahi 6204, Bangladesh; mamun_05ce7@yahoo.com; 3 Department of Civil Engineering, College of Engineering, Prince Sattam Bin Abdulaziz University, Alkharj 11942, Saudi Arabia; r.alyousef@psau.edu.sa; 4 Centre for Future Materials, University of Southern Queensland, Toowoomba, QLD 4350, Australia

**Keywords:** waste glass, alkali-activated cement, aggregate, activator, durability, challenges

## Abstract

Every year, the world is producing around 100 million tons of waste glass (WG), the majority of them are going to landfills that create massive environmental problems. One approach to solve this problem is to transform waste glass into construction materials. Glass is recyclable; however, the melting temperature of the glass is highly dependent on its colour that requires sorting before recycling. To overcome this challenge, many researchers and end-users are using broken glass in concrete either as a binder or aggregates. While significant investigations have done in this area, however, the outcomes of these studies are scattered, and difficult to reach a firm conclusion about the effectiveness of WG in concrete. In this study, the roles of WG and its impact on microstructural and durability properties for both cement and geopolymer concrete are critically reviewed. This review reveals that the amorphous silica in WG effectively participate to the hydration and geopolymerization process and improve concrete microstructural properties. This behaviour of WG help to produce durable concrete against shrinkage, chemical attack, freeze-thaw action, electrical and thermal insulation properties. The optimum replacement volume of binders or natural aggregates and particle size of WG need to be selected carefully to minimise the possible alkali-silica reaction. This review discusses a wide range of parameters for durability properties and challenges associated with WG concrete, which provides necessary guidelines for best practice with future research directions.

## 1. Introduction

The production of concrete requires a significant volume of natural aggregates and non-eco-friendly cement. The extraction of natural river sand and stone chips for concrete construction is increasing day by day, paving us to a shortage of natural resources. The extraction of river sand causes a change in river bed level and hydrological strata, affecting the regular stream directions [1,2,3]. Furthermore, cement production requires substantial energy and emits a large amount of carbon dioxide [4,5]. It was reported that one ton of ordinary Portland cement (OPC) production can release around 0.85 ton of carbon dioxide, which ultimately causes around 5–8% of total emissions in the world [6,7,8]. Thus, dependency upon cement binders and natural aggregates hinders the development of an eco-friendly and sustainable construction sector [9]. Therefore, researchers are always welcomed in finding alternatives to these conventional ingredients.

Globally, around 130 million tons of glass are being produced each year among which approximately 100 million tons are being discarded as waste [10]. Among the WG, only 21% are being recycled [11], and the rest are going to landfill because of the variations in colour and compositions, and being broken and complex. In Australia, according to the statistics of 2019, the WG recycling rate is around 57%, and the rest of them is dumped as waste [12]. Moreover, exporting the WG from Australia is also being banned [12]. Besides, in other countries like UK, USA, Hong Kong, Singapore, the WG recycling rate is less than 50% [13,14,15]. The highest recycling rate is reported in EU (73%) [15]. Thus, a considerable amount of WG is being landfilled each year, which needs to be properly managed. 

As the glass powder containing amorphous silica, thus it can be a perfect substitute for natural sand. Moreover, the high toughness and abrasion resistance nature of glass particles are helpful when used as an effective substitution of natural aggregate in cement and geopolymer concrete. Additionally, the fine glass powder is highly pozzolanic and amorphous, thus can be perfectly introduced into concrete as a partial substitution of binders [13,15]. Most of the previous researches concluded that the fine WG powder helps to increase the pozzolanic reactions in cement-based concrete and contributes to making a densely packed concrete matrix, thus provides high mechanical performances [16,17,18,19]. Additionally, the filler effects and hydraulic characteristics of WG powder also affect the strength development in WG concrete [13,20]. Moreover, glass powder can be effectively utilized as a source of silica, as a precursor or activator solution for geopolymer production. Besides, WG powder can be used as precursors, aggregates, or for developing activator solutions for geopolymer concrete. The WG powder effectively accelerates the geopolymerization process and results in better strength in the final geopolymer concrete [21].

The most common concerning factors are the high alkalinity of WG powder solution and the negative effect of expansion due to the alkali-silica reaction (ASR) gels, which is negatively affecting the strength and durability properties of concretes [22,23]. Although the risk of ASR expansion in geopolymer concretes is less than the cement concrete [24], still it is a concerning point for all researchers.

The durability of concrete is an important parameter that needs to be analyzed before applying it to any environmental exposures. The required durability properties for a typical concrete structure are resistance against shrinkage, chemical penetration/attack, high-temperature variation, freeze-thawing cycle. The dense and compact microstructure is noticed in cement and geopolymer concrete with WG powder [25,26,27]. Thus, the concretes with WG are reasonably durable against any exposure conditions. However, in-depth review in this regard is mandatory to come to any conclusions.

There are some review studies on WG incorporated concrete [28,29,30], but most of those are focused on the mechanical properties of cement-based concrete. In those published review papers, the effect of particle size and amount of glass on the physical and mechanical properties of WG concrete are described. However, the correlation between the role and reactivity of WG within the concrete and the process parameters are not analyzed in those review papers. Also, there is a lack of information and discussion about the durability properties and current challenges of the production and application of WG-based concrete. Besides, the concurrent documentation on the uses of WG in cement and geopolymer concrete will be also helpful for readers and practitioners. This review aims to reveal the durability properties of concrete with WG as a binder, precursor, aggregate in concrete. It includes the current state-of-the-art literature on the cement and geopolymer concretes with WG to reveal the present findings and challenges. Recently the application of WG in concrete is being extended, including precast concrete elements, road paving blocks, marine structures, specially cast foamed concrete, and geopolymer foams [13,31,32]. Therefore, a state-of-art review on the durability properties of cement and geopolymer concretes with WG will pave the way for new researchers and engineers to choose and apply WG concrete for their structures. This study covers the thermal and shrinkage property, performance in chemical exposure, resistance to freeze-thawing effect along with the environmental benefits, and challenges associated with the WG in geopolymer concrete.

## 2. Characteristics of WG in Concrete

### 2.1. Role of WG in Cement Concrete

**Figure 1:**
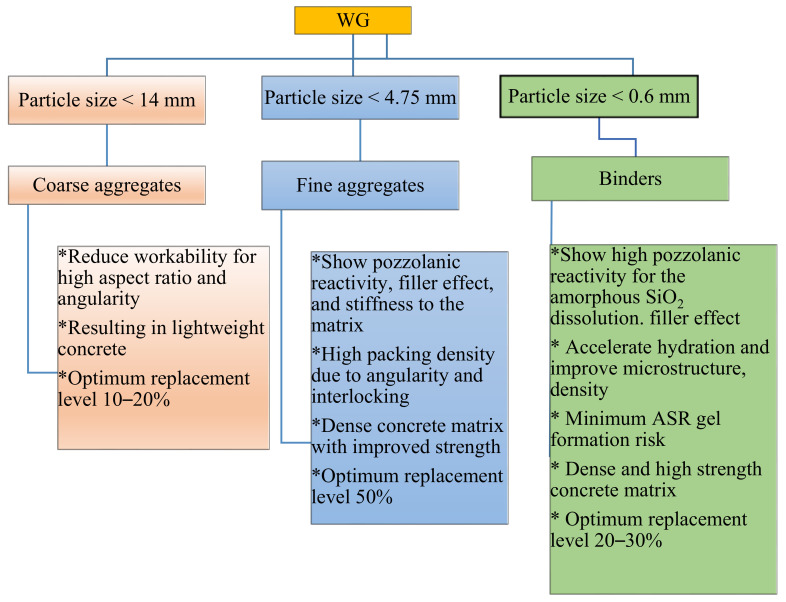
Roles of WG in cement concrete.

Waste glass can be used in concrete as a replacement for binder or substitution of inert materials. However, depending upon the role of WG in concrete and expected outcomes, the typical size of WG particles can be selected. As reported in the literature, the particle size and chemical compositions of WG are the main points that need to be carefully selected during mix design. A typical flow diagram, as shown in Figure 1, explains the size selection and activity of WG in cement concrete.

The main chemical constituents of waste glass are SiO_2_ (71–75%), CaO (8–11%), Al_2_O_3_ (0.95–2.5%), Na_2_O (0–14.5%), MgO (1.6–3.6%), Fe_2_O_3_ (0.3–1%) [20,33]. Given the high SiO_2_ and mostly amorphous nature, WG plays a vital role in concrete, starting from the hydration of binders and up to the final state of strength development. A short induction period is observed for hydration of WG-bases binder, and consequently, the peak heat flows shortly [34,35]. This is an indication of the accelerated production of hydration products (C–S, C–S–H) and a sign of more strength development. According to ASTM C618 [36], materials with 75% pozzolanic index are relatively sufficient to include as supplementary cementitious material, where typical WG powder shows more than 80% pozzolanic index in 28 days age [37]. Observing the amount of reacted Ca(OH)_2_, heat flow during hydration, and final products of hydration, it can be ensured that the WG powder can undoubtedly improve the structure and strength of the concrete matrix [38].

**Figure 2:**
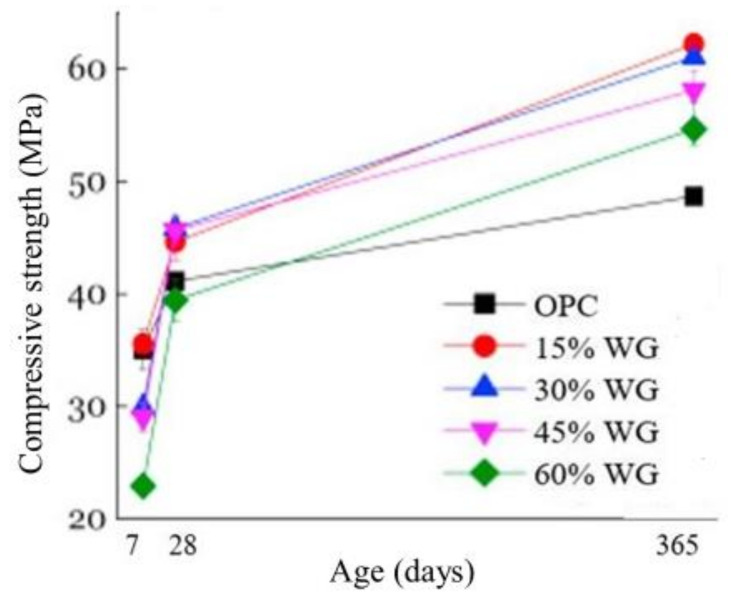
Compressive strength of WGC with WG powder (particles < 120 µm) as SCM [41].

However, to ensure high pozzolanicity, the particle size of WG powder should well below the optimum limit around 38–75 µm [39,40]. Beyond the optimum level of cement replacement, the pozzolanicity and reactivity could be decreased abruptly, as the deficiency of CaO may be started with higher-level replacement, thus resulting in a low amount of CH products [41,42]. Therefore, the inclusion of WG powder should within between 10–30% of the binder, as recommended in previous literature [41,42]. Contrary, it was reported that the early strength development of WG concrete is low. Between 0–21 days of age, WG powder only shows a filler effect in concrete, and after that period, it shows pozzolanic reactivity and participates in the rapid strength development in concrete as shown in Figure 2 [41,42,43]. However, this condition could also generate due to the type of other binders in a concrete and curing condition. Moreover, a high curing temperature (50 °C) can accelerate final hydration products in WG concretes [44]. In general, the reactivity and role of WG in cement concrete are primarily dependents on its particle size, chemical composition, and replacement level. To achieve the best performance, the threshold particle size and optimum replacement level to be designed following the pozzolanic reactivity and ASR guidelines.

### 2.2. Role of WG in Geopolymer Concrete

**Figure 3:**
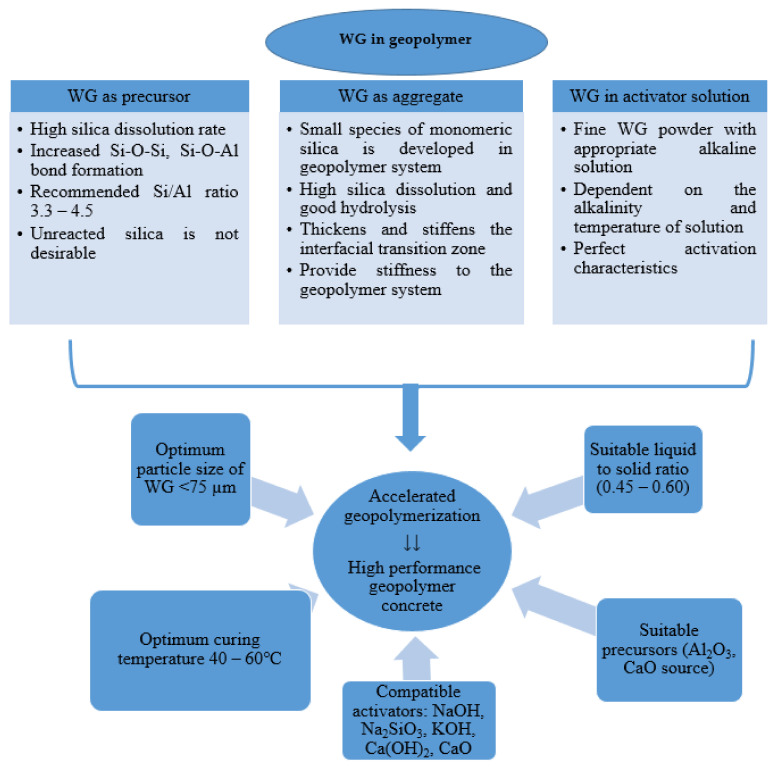
Different roles of WG and the impacts of different parameters on the performance of geopolymer concrete.

A high Si/Al ratio has a significant influence on geopolymerization. With a highly alkaline activator solution, a high amount of silica is dissolved from WG powder in geopolymer concrete [45,46]. Consequently, a significant amount of strong Si-O-Si bond is developed after geopolymerization [47,48]. Some Si-O-Al and Al-O-Al bonds are produced due to the dissolution of alumina from WG and other precursors. The pH of the solution should be maintained above 10.7 to ensure the high solubility of WG [7]. However, unnecessarily excessive alkalinity can hinder the silica dissolution and geopolymerisation. Therefore, the molarity of the alkaline activator should be maintained [49]. Additionally, an excessive amount of silica in the geopolymer system requires a suitable source of Alumina to produce zeolite products. Thus, the recommended range of Si/Al ratio is 3.3–4.5 [50]. A typical flow diagram is shown in (Figure 3), which is self-explanatory to show the effects of WG in the geopolymer system.

WG powder is highly pozzolanic, and its pozzolanicity increases with its specific surface area; thus calcium oxide and hydroxide can be alternative alkaline activators for WG powder-based composites [35]. As it was reported that, the alkali-activated WG paste without additives does not impart hydraulic activity, but the CaO activator can bring the hydraulic property to such paste, which influences strength and microstructure development [51]. Other recommended activators are KOH, Na_2_SO_4_, and Ca(OH)_2_ for WG-based geopolymer concrete [52,53,54,55].

On the contrary, Torres-Carrasco and Puertas [53] reported that there are no significant variations in final products for the variation in concentration and types of activator for WG-based geopolymers. However, this is still unclear and needs to be justified with deep research. Moreover, the final products of geopolymer concrete could be Ca-rich or Al-rich depending on the base material. For high, Ca, or slag-based geopolymer concretes, C–S–H gels are the specific hydration products. On the other hand, fly ash- and calcined-clay-based geopolymer concretes with WG can be considered a low Ca system, and the main hydration products can be N–A–S–H gels [54,56]. However, variations can be seen as per the types of base material and activator, and obviously, these products influence the microstructures and strength of concretes.

Therefore, WG is a suitable source of silica for developing geopolymer concrete by replacing conventional precursors or aggregate. The role of WG and the main parameters shown in Figure 3 are expected to be maintained for high-performance geopolymer concrete.

## 3. Microstructure of WG Concrete

**Figure 4:**
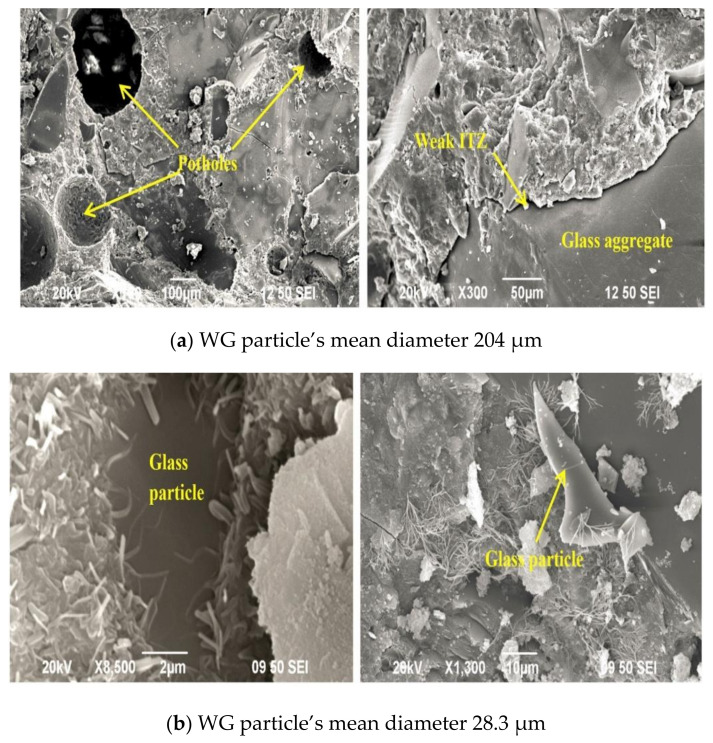
Microstructure and ITZ of cement mortar with WG aggregates [58].

The fine WG powder helps to refine the pore size and divide the ITZ into a very thin layer; thus the density of the concrete increases [57]. However, a weak and porous ITZ can be formed due to the less pozzolanicity of coarse WG particles, and transitional C-H links could be visible (Figure 4a,b) [57,58]. With a 20% WG aggregate (mean particle size around 204 µm), a weak and porous ITZ is visible in cement mortar, and up to 90 days of curing, a significant number of unreacted particles are present [58]. On the other side, the 28.3 µm WG particle produces fibrous hydration products, which make the composite denser and stronger (Figure 4b). Therefore, with the fineness in particle size and curing age, the microstructure of WG concrete becomes denser.

**Figure 5:**
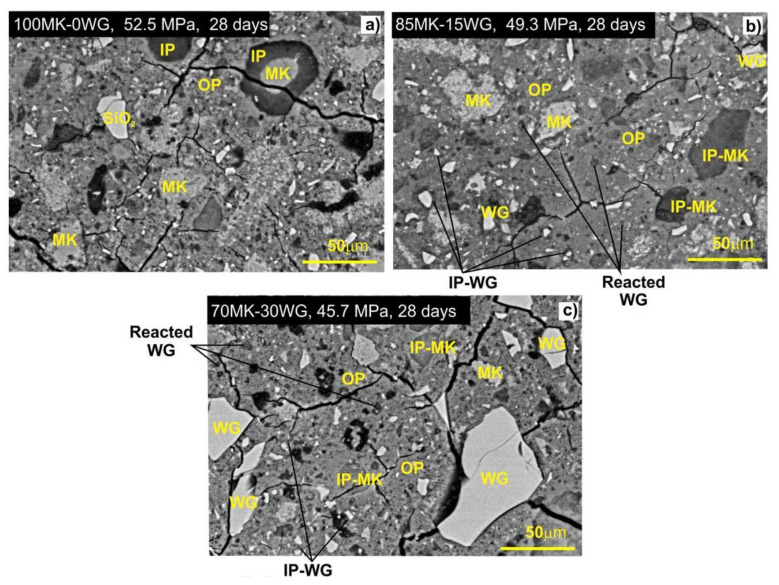
SEM of geopolymers with (**a**) 0%, (**b**) 15%, and (**c**) 30% WG at 28 days (activated with sodium silicate modulus Ms = 1.0 and 12% Na_2_O) [7].

The WG participates in geopolymerization reactions and thus has an impact on the microstructure of geopolymer concrete. From the research conducted by Burciaga-Díaz et al. [7], the SEM view of the geopolymer specimen with 0–30% WG powder and 70–100% metakaolin (MK) are shown in Figure 5. In the SEM image of specimens with no WG, several unreacted metakaolin zones appear as brighter areas and denoted as MK (Figure 5a). Additionally, the inert silica act as a micro-filler and reinforcement in the microstructures of the geopolymer, thus gains strength up to 52.5 MPa at 28 days.

However, after the replacement of metakaolin by 15% WG powder, the matrix density is improved certainly, and there were very low unreacted components left. Thus dark gray spots are less in numbers [7]. Moreover, the development of inner products (marked as IP-MK) is appeared due to the change in final chemical products, such as N-A-S-H geopolymeric gels. Moreover, a difference is evident among the metakaolin consumed products and the main binding phase of outer products.

However, for the increasing amount of WG replacement, the unreacted WG are started to appear in final products, and the width of the internal crack widens significantly (Figure 5c). As it is not any standard results for all the specimens of geopolymer with metakaolin and WG, but still, this can be an example, how the WG addition is certainly changing the micro-structures and micro-pores within the final products. The durability of cement and geopolymer concrete decreases with the presence of micro-pores in the microstructure of hardened products, as it acts as media for absorption and infiltration of solutions/gas from exposures. Thus, the addition of WG up to the optimum limit (30%) is suitable for durability improvement but not beyond it. There are minimal investigations done on the relationship between the internal chemistry of the WG with the base material of geopolymer concrete. Moreover, most of the research only used fine aggregates. Thus, future investigations on these issues can explore more critical findings of WG use in geopolymer concrete.

## 4. The Durability of Concrete with WG

### 4.1. Drying Shrinkage

**Figure 6:**
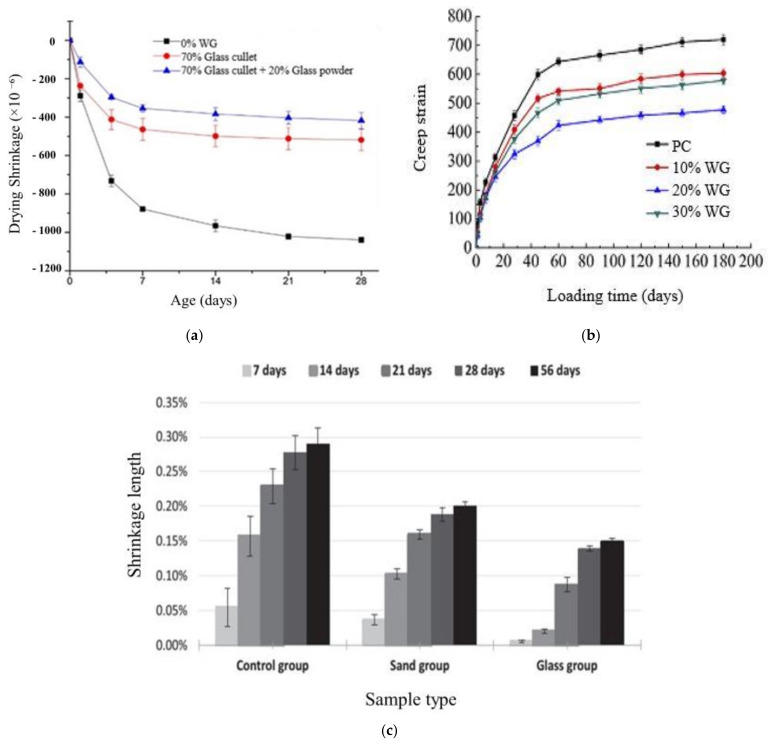
Shrinkage properties of cement and geopolymers concretes with WG. (**a**) Drying shrinkage of concrete with WG [59]. (**b**) Creep strain of concrete with WG powder [42]. (**c**) Drying shrinkage of foamed geopolymer concretes with and without fine WG aggregate [45].

Shrinkage of concrete depends on the type of aggregates, voids, and availability of internal water in concrete. The drying shrinkage of WG concrete is lower than that of plain concrete (PC). As reported in the study of Lu et al. [59], a concrete block with 70% WG aggregates and 20% WG powder possessed 50% less drying shrinkage than PC blocks. This is due to the stiff nature of WG aggregate and its rough surface, which is interlocked with cement paste strongly; thus, shrinkage resistance is enhanced. However, the internal void space in concrete can accelerate shrinkage at high temperature. Thus, finer WG is preferable because fine WG powder shows a filler effect and reduce the voids in concrete. Thus, drying shrinkage reduction by WG powder addition was higher than that by concrete with WG cullet of coarser size [59] (Figure 6a). However, the graded aggregates and WG particles will be more effective to produce dense microstructure and providing the required silica dissolution through the complete hydration stage and will result in reduced shrinkage.

The drying shrinkage is much pronounced by the evaporation of internal water within the concrete pore rather than the surface by thermal drying [59]. Though the water absorption capacity of WG is negligible; thus, the available moisture for evaporation in hydrated cementitious paste within the WG concrete core is lower than that in PC. Consequently, low drying shrinkage occurs in WG concrete. For a similar reason, the creep of concrete with WG powder is generally lower than that of PC at long age. He et al. [42] observed reductions in creep strain by approximately 16.1%, 33.6%, and 19.6% at 180 days since loading, when they replaced cement by 10%, 20%, and 30% WG powder, respectively (Figure 6b). Meanwhile, foam concrete is coarse aggregate-free concrete, in which permissible air voids are left to produce a low-dead load structure. The drying shrinkage is more significant in foam concrete due to the absence of coarse aggregate. The addition of WG fine aggregate or precursors effectively reduces the drying shrinkage in foamed concrete [45,60] (Figure 6a,c).

While talking about geopolymer concretes, major shrinkage occurs at an early age (<90 days) due to the internal water loss from pores and further compaction in unreacted and unpacked base materials. As a result, shrinkage stresses and high strain can arise for highly fine base materials. On the other hand, in WG geopolymer concretes, WG powder acts like a micro filler and refines the pore size; and consequently, the amount of internal trapped water also decreases, which integrally reduces the shrinkage in volume and minimizes the drying shrinkage stress [26]. Besides, the interfacial bond strength of WG particles and the geopolymer binders are very strong [45]; thus the volumetric shrinkage in the hardened products is much lower than the control group without any WG.

For glass-based geopolymer concretes, the rising curing temperature has a positive effect on drying shrinkage reduction because it helps to reduce the portion of evaporable water and resulting in a dense matrix of geopolymer [52]. The drying shrinkage negatively affects the strength and durability of geopolymer concrete, thus needs to be controlled. As the inert aggregate portion does not shrink, thus the higher amount of WG can make the geopolymer concrete more stable against shrinkage [45].

Additionally, shrinkage can occur abnormally when exposed to environmental temperature instead of a uniform laboratory-based shrinkage experiment. Therefore, the long-term serviceability of the WG-based cement and geopolymer concrete under practical conditions needs to be revealed by future deep investigations.

### 4.2. Performance in Chemical Exposure

**Figure 7:**
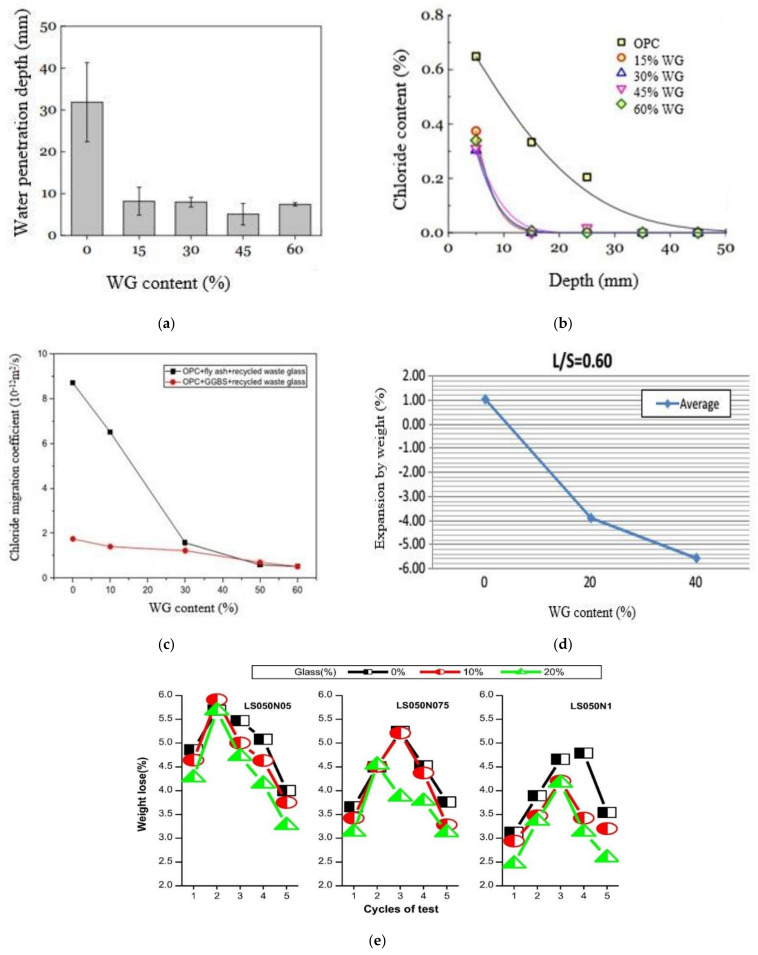
Durability of cement and geopolymer concretes with WG powder [73]. (**a**) Water penetration in WG concrete [41]. (**b**) Chloride penetration with varying WG contents [41]. (**c**) Rapid chloride migration coefficient in WG powder-based mortars [34]. (**d**) Expansion due to sulfate attack in slag-based geopolymer concretes with WG powder [69]. (**e**) Weight loss in geopolymer concretes with WG powder [73] (LS = liquid to solid ratio, N = % of activator).

The most important durability parameter of concrete is the penetration and absorption of water and chemical into its pores. Given the filler effect and pozzolanic reactivity of fine WG powder, the density of WG-based concrete increases, and the porosity and pore connectivity decreases. Thus, the water and chemical absorption and penetration into WG-based concrete are generally less than those of PC up to an optimum replacement level of cement and aggregates (Figure 7a [41]). In addition, the impermeable nature of WG powder [19] can be another reason for the reduction in the water absorption of WG concrete. Thus, the chemical penetration resistance in WG concrete is significantly higher than that in PC.

A rapid chloride penetration test on WG concrete was performed by Hilton et al. [25], who observed a low chloride permeability of concrete with mixed contents of WG powder as a partial replacement of cement. Moreover, Omran and Tagnit-Hamou [61] tested WG concrete for up to 365 days of age to investigate chloride ion penetration and observed 64% improved resistance to chloride penetration in concrete with 20% WG powder. Du and Tan [41] achieved 77% reduced chloride penetration depth, and 92% lowered chloride migration coefficient for concrete with 60% cement replaced with WG powder (Figure 7b). A similar concept was recorded from the study of Wang et al. [62]. The primary cause of this increasing resistance is the densely packed and minimally porous internal structure of concrete, which helps reduce the permeability of any chemical solution. Additionally, fine glass powder disrupts the pore connectivity within WG concrete, and it works to reduce the chloride penetration. Friedel’s salt formation was also observed with the aluminium phase in WG concrete, contributing to resisting the negativity of chloride penetration [63]. A typical graph (Figure 7c) on the rapid chloride migration coefficient of glass-based mortar confirms the high performance of high-content WG powder-based binders in chloride media.

Authors [25] also reported a good resistance to sulphate attack in WG concrete with 15% mixed WG. The test conducted by Tayeh et al. [64] revealed reliable sulphate resistance of cementitious mortar with 10% WG powder compared with conventional mortar. However, concrete can be vulnerable to sulphate attack, as it degrades the hydration products, decalcifies C–S–H products, and pronounces leaching, which is more vulnerable to magnesium sulfate sulphate attack than sodium [65]. The addition of high pozzolanic WG powder transfers the CH products into C–S–H products, which enhances the durability against the sulphate attack.

Carbonation is another effect developed after the penetration of carbon and oxygen into concrete pores, connected with the corrosion of steel rebars within reinforced concrete (RC) elements [28]. Carbonation depth in concrete depends on the relative pore size in concrete and environmental humidity because the diffusion of CO_2_ accelerates with these factors. A considerable amount of WG causes porous WG concrete; consequently, increased carbonation was reported in previous research [66].

Beyond a certain optimum level of WG powder substitution, the chemical penetration in WG concrete is generally increased. This increase is due to the agglomeration of WG powder and the low production of C–S–H, resulting in porous and low bonding in WG concrete. Increasing pore and loose bonds can easily act as media for chemical penetration. When WG powder is used as a replacement for cement in excess (>50%), the secondary C–S–H products transform into M–S–H gels in magnesium sulphate media [67]. When WG aggregate contacts with NaOH media, the ASR expansion is increased momentarily. The internal micro-cracks are the space providers for the ASR gel formation. The dissolved silica and sodium ions within these micro-cracks lead to disruptive diffusion and expansion, lower concrete’s durability. The tiny internal pores and micro-cracks can be filled by ASR gel expansion rather than the large pores. The ASR gel formation cannot initiate within the large pores and internal cracks because of the unavailable pore solution [60].

Geopolymer concretes generally show high durability against chemical attacks. As observed from the study of Torres-Carrasco et al. [68], WG powder reduces the porosity in the hardened composite of geopolymer concretes, and thus, the chloride penetration resistance increases. Wang et al. [69] observed reduced weight loss due to sulphate attack in geopolymer concretes with up to 40% WG powder replacing slag (Figure 7d). Sulphate resistance increases with increasing liquid-to-solid ratio, but an optimum condition must be maintained to ensure a high rate of geopolymerization and high packing density. As shown in Figure 7e, for liquid-to-solid ratio of 0.5, the geopolymer concretes with 10% glass sand are shown a weight loss due to a sulphate sulphate attack at about 2.94–5.92% in the first three consecutive cycles. For a 20% replacement level, the loss in weight is much lower than the control specimens. Thus, it is satisfactory. However, the weight loss observed in the WG-based geopolymer concretes activated with 0.5% alkaline solution is higher than that of the 0.75% and 1% solutions. This is because the activator solution with lower alkalinity cannot break down the complex slag structure rapidly compared to the highly alkaline solution. Therefore, a porous geopolymer concretes matrix is resulted, and consequently, sulphate resistance of that concrete decreases.

Additionally, the increasing porosity can lower the resistance to the chemical attack in WG powder-based geopolymer concretes, which may be attributed to unreacted silica. Unreacted WG powder can be the reason for the deterioration in sodium aluminosilicate bonding and leaching in mild- to high-concentration acidic media [70]. The deterioration rate depends on the type of acidic media. For example, geopolymer concretes with WG powder disintegrate more in sulfuric acid media than hydrochloric acid media [16]. The formation of gypsum crystals within the WG-based geopolymer concretes produces internal stress that causes internal cracking and spalling, and progressive durability deterioration occurs in sulfuric acid media [70].

Moreover, the leaching of unreacted alkali is a common problem in geopolymer concrete, resulting from activation with a highly alkaline solution. The molarity of the alkaline actor should be compatible with the amount of WG in geopolymers; as the content of WG increase, high alkaline media is required to activate entirely. However, for low replacement levels, excessive alkaline solution is more vulnerable regarding high alkali leaching. There is a lack of details investigations on the optimum level of WG and proper alkalinity for the WG-geopolymer system; thus, future investigations on this issue are required.

High curing temperature (40–60 °C) and long curing periods are recommended for stable WG-based geopolymer concrete with high mass stability and minimal leaching [52,71]. However, chances of efflorescence in WG-based geopolymer concretes are high in a humid environment because of high alkalinity. To balance the high alkaline content along with the dissolved silica, a suitable alumina source is required, which will result in lowering the efflorescence risk.

Besides, silica depolymerization may occur due to the removal of physically bound water [54]. The addition of aluminium- and calcium-rich base materials during geopolymer preparation and hot-water curing are effective methods to minimize deterioration risk [54]. The moderate alkalinity of the activator can set substantial ions to form confined geopolymeric gels and consequently improve the durability against leaching [6]. The dense microstructures and less pore connectivity assure the immobilization of ions, as the penetration and removal of physical water are prevented. Additionally, uniform, and dense micro-structures are a result of less unreacted particles. Thus, it is lowering the reactivity of the leaching solution and prevents the contaminants to leach out. The WG in which mercury and lead contents are present show high mobility in geopolymers and tends to leach than the other contaminants [72]. However, the mobilization of the contaminants in the waste materials-based geopolymers is not fully clear. Therefore, details and deep investigations are a major demand.

However, there is minimal data, and very few investigations were done on WG-based geopolymer concretes to evaluate the durability against chemical exposure conditions. Therefore, this review finds a lack of details investigation on the durability of glass-based geopolymer concrete at the current state of the art. Thus, detailed investigations are needed to find the appropriateness of WG-based geopolymer application in a harsh environment, especially in chloride and acidic medium.

### 4.3. Freeze-Thaw Resistance

**Figure 8:**
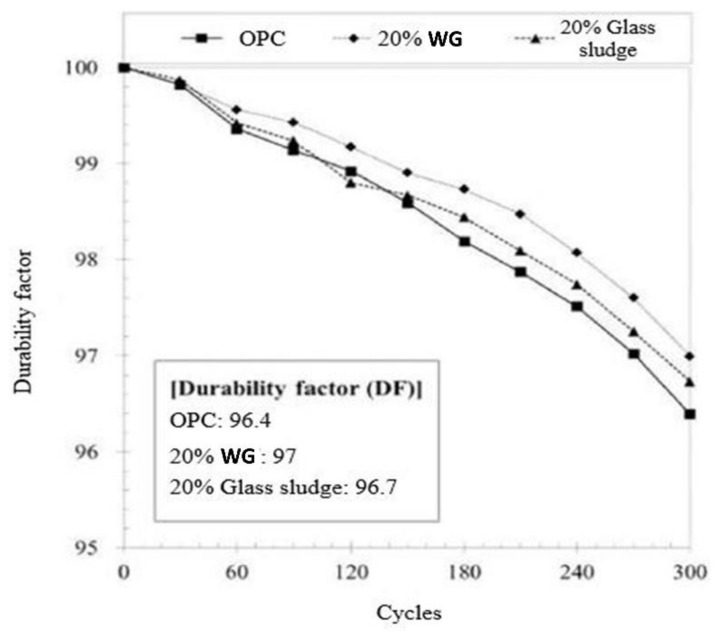
Durability factor of WG concrete against freeze-thaw action [27].

Porous concrete is more susceptible when exposed to freeze-thawing cycles. The stress is generated due to the freezing cycles that cause internal micro-cracks in the concrete and create an additional path to penetrate chemicals and water into concrete. Thus, dense matrix and less susceptible aggregates are preferable. High resistance to freeze-thaw cycles of WG-based cement concrete has been reported in previous researches [17,74]. The freeze-thaw resistance of WG concrete was tested by Lee et al. [27], and they observed approximately 24% better resistance to scaling due to 50 freeze-thaw cycles in concrete with 20% WG powder compared with PC. As represented in Figure 8 [27], the durability factor of WG concrete is much higher than that of PC for freeze-thaw action up to 300 cycles. The durability factor is presented by DF = P_n_ (N/M), where P_n_ is the relative dynamic modulus of elasticity at n cycles (%), N is the smallest number of cycles at which P_n_ reaches the minimum threshold value for stopping the test or at which exposure is to be terminated, and M is the designed number of cycles at which the exposure is to be terminated. With the increasing number of cycles, the durability factor decreases, but in every cycle, the durability factor for WG added sample is higher than the others, as shown in Figure 8. High pozzolanic characteristics and filling effect of fine WG powder make concrete dense, which improves the dynamic modulus of elasticity and durability. Thus, Lee et al. [27] found that fine WG powder is superior to glass sludge in terms of frost resistance. Besides, fine WG powder offers compactness in microstructure and provides high density; thus, the pore volume inconsiderably contributes to the deterioration due to the freeze-thaw effect. Free water content induces excessive stress during freeze-thaw cycles. When stress is generated from the freezing cycle, a certain degree of stress relief can be expected due to the air voids within the concrete, which consequently reduces the cracking [75]. Meanwhile, the free water induces from the thawing cycles can cause bulking of unreacted sand aggregates, but when a certain amount of sand is replaced by WG powders, it can resist bulking. Thus, the stress due to bulking and deterioration of hydrates in concrete from water gain is resisted. Therefore, WG concrete can be used in the construction of marine structures and cold-weather regions. However, no chemical decomposition is reported in the literature, thus future analysis is required on this issue

Dense WG-based geopolymer concretes offer high resistance to freeze-thaw cycles. WG powder influences the formation of strong Si–O–Si link, and deterioration due to chemical decomposition is minimal [47,48]. Additionally, the dense micro-structure of geopolymer concrete with WG powder can resist the considerable stress developed in freezing cycles. However, any micro-cracks in hardened composite could be the reason for water penetration, destructively reducing the durability against frost action. The relationship of porosity and strength with freeze-thaw resistance depends on several other factors, such as aggregate size, WG powder substitution level, and WG powder particle size, which should be investigated further. At the current state of the art, conclusions cannot be made on freeze-thaw resistance of glass-based geopolymer concretes because of the lack of adequate research data and analysis.

### 4.4. Electrical Resistivity

**Figure 9:**
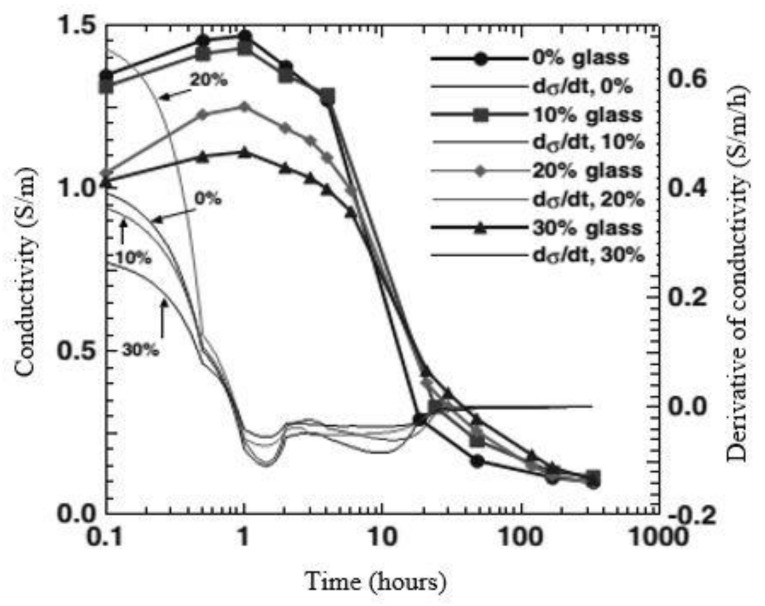
Electrical conductivity of cement–WG powder paste [77].

High bond strength and less porous microstructure of the WG-concrete matrix effectively resist electrical charges passing through it. Porosity, pore connectivity, pore solution, and ion mobility in concrete are the main factors that control electrical resistivity. The addition of WG with cement-based composite improves electrical resistivity, which may be attributed to the change in chemical composition within the pore solution because of the high alkali content [76]. Schwarz et al. [77] experimented on the electrical conductivity of cement paste with WG powder. They observed less conductivity in cement and WG powder paste compared to the control, as represented by Figure 9. At the early age of mixing, the minimal content of ions and the substitution of cement by WG powder caused a reduction in electrical conductivity. After the dissolution and induction period, the conductivity decreased significantly with time due to the production of final products. Fine WG powder reduces pore connectivity and interrupts the mobility of ions; thus, conductivity is reduced. When the porosity of concrete and interconnection among pores is high, concrete’s electrical resistivity decreases.

It is already proven that geopolymers possess high electrical resistivity. The alkalinity of the activator solution and the base materials are the main controlling parameters for the resistance to electrical conductivity. As reported in researches [78], fly ash-based geopolymers offer higher electrical resistivity, but the molarity of alkaline activator solution harms this resistance. Glass is non-conductive, and thus, it acts as a barrier to charge flow. Increasing the slag replacement ratio by WG results in more resistance to electrical charge pass. The interconnected pore is not desirable because these provide a path for charge flow. However, there is no such research done on the electrical properties of glass-based geopolymer concrete to investigate the conductivity; thus the variation cannot be certainly concluded. Therefore, significant research on this issue is a prime need to evaluate the durability of glass-based geopolymer concretes.

The typical durability properties of WG concrete observed from previous research are listed in Table 1. This review states that WG concrete is durable in severe chemical exposures and can be used to construct chemical exposures. As reported from previous research, 50–92% reduction in chloride penetration, 300% improvement in electrical resistivity and significant improvement in resistance to sulphate attack and carbonation problems can be achieved after the inclusion of WG in concrete. However, precautions should be taken to achieve optimal results. This review reveals a lack of details investigations on the durability properties of WG-based geopolymer concretes. Therefore, future investigations are required for the role of the chemical composition of glass, gradation of particle size, and the level and type of replacement and their effect on the durability of geopolymer concrete.

### 4.5. Thermal Properties

**Figure 10:**
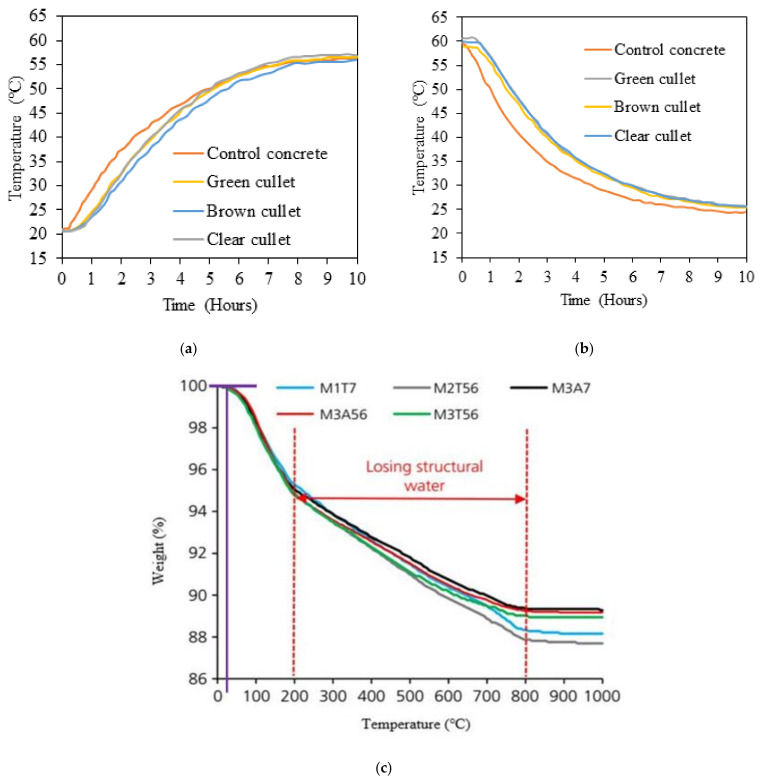
Performance of glass-based cement and geopolymer concretes with varying temperatures and times [84]. (**a**) Variation in WG concrete temperature with varying environmental temperatures (rising) and time [83]. (**b**) Variation in WG concrete temperature with varying environmental temperatures (falling) and time [83]. (**c**) Weight loss in geopolymer concretes with varying temperature [84] [M1 with no glass, M2 with 10% and M3 is with 20% glass powder (varying curing condition)].

The thermal conductivity of concrete depends on the type and size of aggregates, density of matrix, and the size and content of air voids [82]. The inclusion of WG in concrete effectively alters the density and porosity of hardened composite; thus, it also has a great influence on thermal conductivity. WG aggregate possesses lower specific heat compared with natural sand, and WG concrete shows greater stability in temperature variation. Poutos et al. [83] observed a lower variation in internal temperature within WG concrete than that of PC when the surrounding temperature increased from −20 °C to 60 °C. The rise and fall of temperature within PC are comparatively faster than the rise or fall of temperature within WG concrete for the same surrounding temperature variation, as shown in Figure 10a,b [83]. This condition is attributed to the porous structure of WG concrete and the low specific heat of WG, which lowers the temperature flow inside the concrete core. Thus, the WG is suitable to develop foamed composites and autoclaved aerated concrete as insulation materials for infrastructures.

In geopolymer concretes, the addition of WG powder causes a reduction in thermal conductivity for the minimal interconnections in the internal pores, thereby preventing the temperature flow inside the composite [45]. The coefficient of thermal conductivity of WG concrete was slightly higher than the PC in the observation of Andiç-Çakır et al. [23], in which the conductivity increased with an increase in the particle size of WG aggregates. This finding was attributed to the densely packed matrix and low air voids within the hardened composite. A similar conclusion was observed on geopolymer concretes with WG powder from the study of Wang et al. [69]. The high packing density caused a slight increase in the thermal conductivity for geopolymer concretes with 40% WG powder, but they concluded that the effect of liquid-to-solid ratio was much more than the effect of WG powder addition. The density and porosity vary with WG powder content, and thus the thermal conductivity also varies. Hajimohammadi et al. [45] developed geopolymer foam for insulation purposes using 30% WG fine aggregates. They reported 77% stronger geopolymer foam at 600 kg/m^3^ density when 30% WG is used, and the thermal conductivity was around 0.15 W/mK. The lowest pore connectivity was observed in the WG-based geopolymers compared to the sand-based or control geopolymer. However, the porous structure within cement concrete or geopolymer concrete is undesirable because it is related to mechanical strength reduction.

The glass transformation temperature is 600–800 °C. At a high temperature beyond this indicated value, significant changes occur in the glass and WG concrete’s behavior. The contribution of melted WG powder to the residual strength of concrete after exposure to glass melting temperature is sufficiently high [58]. The melted WG powder can heal the micro-cracks within concrete after being cooled and increase the residual strength compared with ordinary concrete.

The mass loss in glass-based geopolymer concretes at high temperatures (approximately 200–600 °C) is more than that in sand-based geopolymer concretes [21]. The condensed nature of WG powder-based geopolymer concretes offers high resistance to elevated temperature. Most strength losses occur due to the evaporation of structural water [85]. This phenomenon is caused by the characteristics of hydration products in WG powder-based geopolymer concretes. Chemically bound water content is high for condensed geopolymer concretes [86]. At room temperature to approximately 180 °C, the physically absorbed and surface water evaporates, and mass loss is most significant at this range of temperature [86,87]. With rising temperatures, the dehydroxylation of geopolymer products is started and continued at approximately 350 °C temperature [86,87]. At a temperature of approximately 600 °C, chemically bound water loss occurs and may result in densification but does not show stability with time [86]. Geopolymer concretes show distinct behavior for different raw materials and activators at elevated temperatures, depending on the nature of final reaction products; high-concentration WG powder-based composite may undergo melting. The decomposition of silicate and carbonate products occurs at a temperature of approximately 800 °C [84], as shown in Figure 10c.

**Figure 11:**
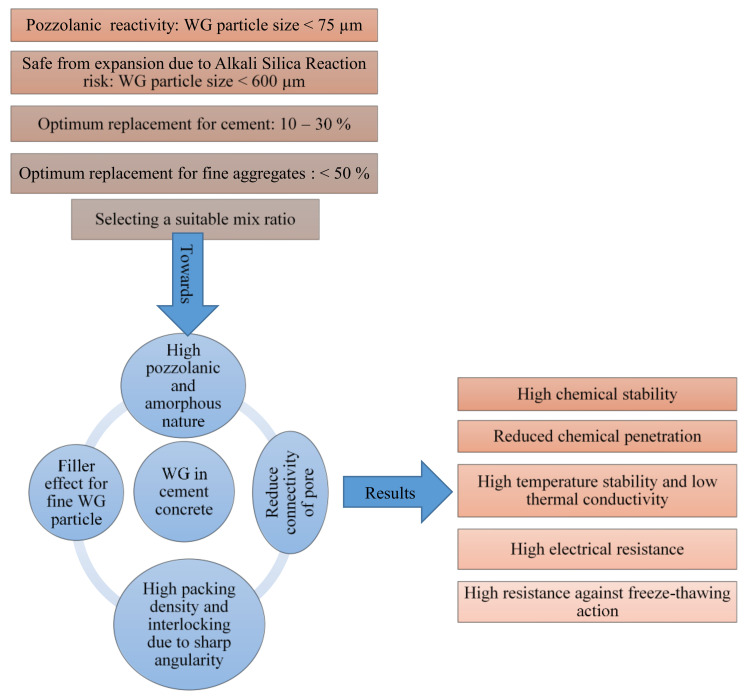
Influences of WG on the durability of concrete.

The high shrinkage due to the softening of glass and more ASR expansion could resulted in with the increasing temperature in WG-based cement and geopolymer concrete [40,88]. These issues are not clear in literature. Therefore, extensive future research are required on this major concerning point regarding the negativity of high temperature in cement and geopolymer concrete with WG. A typical representation is shown in Figure 11, which clearly describes the effects of WG particles on the formation of the structure of the cement concrete matrix and towards the improvement of durability. However, the durability of WG-based geopolymer concrete is still under investigation and needs more significant documentation to finalize the relationship of WG with the microstructure development and durability issues. Additionally, though a high amount of WG particles produce porous concrete, high thermal insulation can be obtained by WG-derived composites. Foam made from WG can be perfectly used for thermal insulation and durability purposes [89,90], but its mechanical strength is insufficient for structural application. Therefore, application of the alkali-activation technique, the incorporation of additives, and fibres, an advanced glass-foamed composite can be made, which simultaneously will resist the service load and imparts the durability and thermal insulation of the structure. Therefore, detailed investigations on this topic are now a major demand.

## 5. Environmental Benefit of WG Concrete 

**Figure 12:**
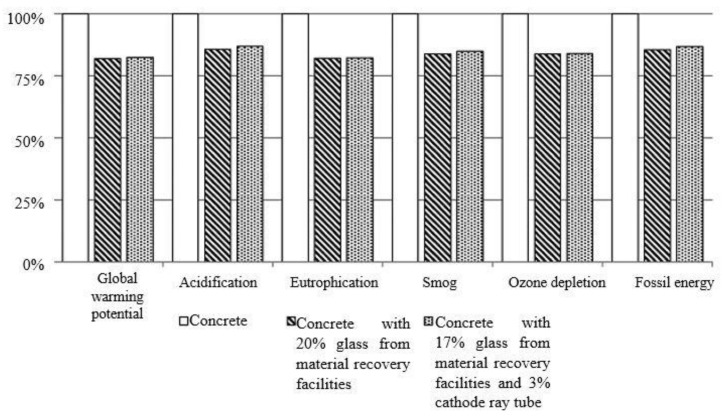
Environmental impact of PC and WG concrete [25].

Recycling of WG as a construction material simultaneously reduces solid waste management problems, demand for landfills, and carbon footprints and problems on resource preservation [14]. The environmental impacts of PC and WG concrete were investigated by Hilton et al. [25], and they revealed 13.2% reduced environmental impacts for WG concrete compared with PC (Figure 12). In addition, a 20% reduced global warming potential in WG concrete is a good contribution to environmental sustainability compared to PC. 

**Figure 13:**
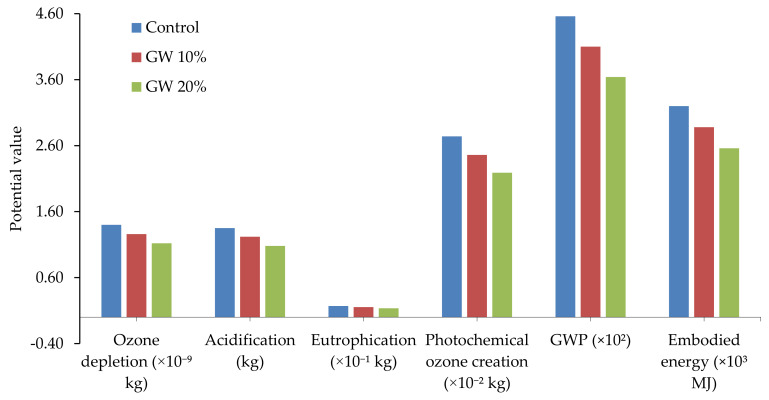
Environmental impact of WG recycled cementitious mixture [91].

Glass-based cement produces approximately 0.17–0.42 gCO_2_/gWG powder, resulting in up to an 83% reduction in CO_2_ production compared with OPC [51]. Similar results were obtained from the study of Patel et al. [91], who reported that eutrophication, ozone depletion, the energy embodied, acidification rate, photochemical instability, and WGP reduce with the increasing content of WG in a cementitious mixture (Figure 13); a significantly high environmental benefit is ensured in comparison with control groups. These studies represented the environmental benefit of WG concrete. The recycling of WG could be a major source of raw materials and can facilitate saving natural resources and nature. The total solid waste management system will be benefitted, and a healthier environment can be expected in the future. However, the long-term serviceability, carbon footprints, environmental impact assessment is needed to be done on WG base cement and geopolymer concrete to rate this composite as a sustainable material.

## 6. Challenges in WG Concrete and Remedies

### 6.1. ASR Expansion in WG-Based Concrete

One of the major challenges of WG concrete is the presence of high silica, and alkali content in glass and cement causes the ASR, which could cause expansive gel formation [22,23,92]. The ASR expansion is accelerated with the presence of Na and K ions [92]. The ASR gel produces expansive stresses along the reaction zone, which may cross the limit of the tensile strength of concrete; thus, cracks can be developed. Thus, an additional pre is created for penetration and absorption of the external solution and consequently deteriorates the durability.

However, the risk associated with ASR gel formation can be minimized using finer WG powder instead of coarse glass aggregates. The critical particle size of WG powder is margined by researchers as 1–1.18 mm [39,93]. However, some of the literature marked 0.6 mm particle size as a safe limit [94]. For example, the replacement of 70% fine aggregates with 36–50 µm particles of WG powder in concrete did not exhibit any harmful ASR expansion in previous research [95]. Moreover, researchers concluded that the glass sand particle size below 4.5 mm without any surface cracks does not show any expansive ASR gel formation for up to 40% sand replacement level [96]. Micro-cracks in the WG particle are not desirable, as they create pores and store solutions for future reaction, and consequently, ASR reactivity increases. This ensures that only particle size is not solely affecting the ASR risk; some other factors like the content of WG, nature of cement and aggregates, mix ratio, the water-cement ratio of the concrete mix also influencing ASR gel formation. Therefore, depending upon the chemical properties of WG and maintaining an optimum level of replacement and particle size, ASR risk can be minimized. However, properly graded WG powders can enhance the density and reduces the ASR expansion. Besides, the presence of lithium ions suppresses the expansion by changing the ASR gel composition [97,98].

The risk of ASR expansion in geopolymer concrete is observed less than the ordinary cement concrete. As reported in the literature, the high silica dissolution and alkali activation are effective for geopolymerization and development of dense microstructure of geopolymer concrete. Thus the major part of alkali ions is being balanced through ASR [21,51]. A comparison made by researchers showed that the geopolymer concrete might undergo only 5% ASR expansive gel formation compared to the cement concrete [24]. The alkali present in geopolymer raw mix works with activator solution and forms crystalline silicate products and zeolites in different forms; thus the risk of expansion reduces. Depending upon the alkalinity and nature of the precursor, the nature of final products varies, but intermediate products developed during geopolymerization can exchange ions with excess alkali. Thus, the ASR expansion risk cannot be ignored completely.

The challenge of minimizing ASR expansion should be carefully considered during the mix design and application of WG concrete. Expansive alkali-silica-based products can be transferred to rigid and stiff products in concrete, which will also be effective in enhancing the strength of the concrete. However, investigations on this topic are still ongoing in several fields. For example, Lee et al. [99] explained that using borosilicate glasses with additives in the cementitious mixture could reduce ASR expansion, improve strength, and be useful for neutron shielding. Other measures can be taken to reduce ASR expansion, such as the use of polyester resin to remove alkali, and stabilized by fly ash, metakaolin, silica fume, and blast furnace slag [23,40,100]. The presence of high content of glass stabilizers, such as CaO and MgO, and minimal glass modifiers, which may be the oxides of Na, K, and Pb, can lower the amount of formed ASR gels [92]. When using lithium treatment, a considerable reduction in ASR expansion can be achieved. Lithium compounds, such as lithium carbonate, lithium hydroxide, lithium nitrate, lithium chloride, and lithium fluoride, have been used for a long time [29]. The presence of microcrystalline lithium silicate precipitate reduces the dissolution rate of silica and stabilizes the amorphous silica to prevent reaction with alkali [29].

Moreover, the Ca/Si ratio must be within the threshold value because a low value of Ca/Si in the concrete mix causes considerable ASR expansion [29]. Thus, using a suitable Ca source simultaneously improves the pozzolanic reactivity and reduces the ASR expansive gel formation in concrete. Eggshell waste contains calcium carbonate and Ca-rich waste. Thus, eggshell powders can be used as a source of Ca in WG-based cement and geopolymer concrete. However, there is no experiment done to incorporate this eggshell waste, thus, its applicability needs to be investigated. Other recommended techniques for ASR mitigation are presented in Table 2.

### 6.2. Low Adhesion between WG and Cement-Paste

Low adhesion between cement paste and WG is another major issue, which is a reason for strength reduction in concrete [101]. Porous and weak ITZ can develop from the weak adhesion of WG powder and binder paste [102]. The main causes of low adhesion are the smooth surface of WG and micro-crack within particles [103]. A rough surface of WG can provide interlocking with cement paste, but excessive roughness could generate a porous structure. Well-graded glass particles are suitable for high packing density. The pretreatment of WG using heat or polymer resin can increase bond strength with bonder paste, which needs further investigation to be established. Other problems that could hamper the strength development in WG concrete are also listed in Table 2. As discussed in the previous sections, the mechanical properties and durability of WG concrete can degrade if micro-cracks are present in WG. The preparation of WG powder and aggregate should be under supervision.

### 6.3. Other Challenges and Research Gaps

Another challenge of WG-based geopolymer concrete is incorporating a suitable activator and precursor for high volume WG geopolymer. There is no data available in the current state of practice to activate the WG-powder binders with a suitable binder. However, the alkali activation of WG powder is good and compatible but needs an additional additive to gain certain strength, as metakaolin, fly-ash, or slag. Thus, for high volume WG binder activation and full-strength development, a suitable activator is needed. Similarly, using the WG-derived activator is still under investigation, which needs to be established.

However, in the current state of the art, there is a need for investigations on the durability properties of waste glass-based geopolymer concrete, as in current research gaps are being revealed through this review. As reported in previous researches that the geopolymer concretes can stabilize heavy metal ions with their complex geopolymeric networks [6], but no significant researches have been developed to support the claim that the WG powder-based geopolymer concretes can do the same. Additionally, the durability of glass-based geopolymer concrete needs to be investigated against acidic and chloride media, as there is no such research contribution found in the current state. Therefore, to incorporate WG into a geopolymer system and widen its application in construction industries, detailed and deep investigations must establish conclusions and guidelines. Meanwhile, the application of coarse aggregates in geopolymer concrete along with the glass aggregates needs to be adequately investigated, as there are no current data available to bring any recommendations.

## 7. Conclusions

This review includes a critical discussion on the current research progress of cement and geopolymer concretes containing waste glass. The durability of concrete is a major concerning point, where different degrees of durability may require each type of concrete depending on their exposure conditions. However, WG addition significantly altering the microstructure and product characteristics of concrete; thus its durability needs to be investigated broadly. Current research progress is not sufficient to address significant guidelines and examples of durable WG-based concrete. The following conclusions are drawn from the state-of-the-art review:

- The waste glass acts as a rich source of silica in concrete. Thus the pozzolanic activity increases, hydration product formation increases, and microstructures get improved after the addition of fine WG in concrete. Additionally, the silica dissolution in the geopolymer system also increases due to the presence of fine WG powder and consequently improved geopolymeric reaction. To optimize the silica dissolution and pozzolanicity, the optimum particle size of WG must be maintained as recommended around 38–75 µm.
- WG powder does not hold free water in the internal pores of the concrete and minimizes the pore connectivity. Thus a lower drying shrinkage occurred. Additionally, curing with raised temperature is effective to reduce the shrinkage and improve the micro-structure compactness.
- The high pozzolanic reactivity and filler effect of fine WG powder result in a high-performance composite with high durability against water, chloride, and sulphate penetration and adverse effect of any chemical attack. Concrete’s resistance to acid attack and carbonation is also improved. Besides, electrical charge flow, the thermal conductivity of glass-based concrete and geopolymers are reduced due to the addition of WG.
- Very limited research has been conducted on the durability of WG-based concrete; thus the recommendation for optimum level of WG inclusion replacing binders or aggregates in concrete remains an open research question. However, based on current knowledge, it is estimated that the optimum level of binder replacement could be around 20–30%, and this range is approximately 30–50% for fine aggregate. Beyond the optimum level of replacement, a porous concrete matrix will result in lower durability.
- The most critical issue of glass incorporation is the ASR and expansive gel formation within concrete. This issue is less critical for geopolymers compared with cement concrete. The ASR expansion can be minimized by using fine WG powder (<75 µm), replacing cement instead of aggregates, and adding recommended by-products, such as silica fume, fly ash, and slag optimum level of around 10–30%.

This review reveals that no significant investigations have been done on the durability of geopolymer concrete with WG. Additionally, there is a lack of details of investigations on the chemical attack of concrete with WG. Therefore, it is highly recommended to investigate the durability of the cement and geopolymer concrete with WG by considering all possible exposure conditions. Furthermore, while ASR is critical for cement concrete containing WG, the behaviour could be different for geopolymer concrete. Therefore, a comparative evaluation between cement and geopolymer concrete having similar physical and mechanical properties would be interesting for future investigation on ASR.

## Figures and Tables

**Table 1:** Durability performance of WG concrete.

WG Type	Replacement Level	Durability Performance Compared with Control Specimen	Remarks	Ref.
Soda-lime glass bottles (<4.75 mm)	0–100% fine aggregates	Enhanced resistance to chloride penetration Approximately 12% reduction in drying shrinkage for 75% WG powder ASR reactivity in green and brown glasses is negligible.	Micro-cracks in glass sand and weak bond with the cement paste resulted in low mechanical performance.	[79]
Mixed types (100–600 µm)	0–50% cement	46.7% reduction in chloride permeability for 10% replacement at 28 days of age	Fine WG powder reduced the ASR expansion risk and improved durability.	[80]
Soda-lime glass bottles	0–60% cement	91% reduction in chloride diffusion rate and 92% reduced migration coefficient for 60% WG powder 30% replacement level is optimal for the lowest porosity	Dense interfacial transition zone (ITZ) formed due to fine WG powder	[41]
Glass bottle (fineness of 400 and 600 m^2^/kg)	30% cement	42–50% reduction in chloride permeability for 30% replacement at 28 days of age 206–308% improved electrical resistivity after 28 days of curing 87% less expansion due to sulphate attack at two months of age	Durability performance increases by increasing the fineness of WG powder.	[76]
WG (<14 mm for coarse aggregates and <4.75 mm for fine aggregates)	10–30% fine aggregates and 5–15% 10 mm coarse aggregates	Approximately 6% reduced chloride penetration observed for 30% fine glass sand and 15% coarse WG aggregates ASR expansion and drying shrinkage were below the standard limit.	High content of WG aggregates may cause segregation in concrete.	[81]

**Table 2:** Challenges and remedial measures.

Challenges	Techniques for Optimization	Ref.
ASR expansion in WG-based concrete	Use of polyester resin to remove alkali Use of borosilicate glasses Use of metakaolin, fly ash, and silica fume Lithium treatment of WG Inclusion of blast furnace slag	[23,29,40,60,99,100]
	Inclusion of nano-materials and source of calcium can lower the ASR gel formation Rapid hardening and self-curing concrete can develop to reduce the porous form of the concrete matrix	Author’s suggestion
Low adhesion between WG and cement paste	Well-graded and fine WG is required Rough and angular surfaces of WG must be produced Prevention of formation of micro-cracks in WG during grinding Short textile fibers derived from waste textile can be used to enhance the bridging and bonding	Author’s suggestion
Suitable activator and compatible precursor for WG	WG is a pozzolanic material, thus can be activated by CaO, KOH, Na_2_SO_4_, and Ca(OH)_2_, but final products could be different	[51,52,53,54,55]
	The inclusion of eggshell waste powder can be a source of Ca in WG-based precursor	Author’s suggestion

## Data Availability

Not applicable.

## References

[1] Bahoria B.V., Parbat D.K., Nagarnaik P.B. (2018). XRD Analysis of Natural sand, Quarry dust, waste plastic (ldpe) to be used as a fine aggregate in concrete. Materials Today: Proceedings.

[2] Islam M.T., Islam M., Siddika A., Mamun M.A. (2021). Al Performance of rubberized concrete exposed to chloride solution and continuous wet–dry cycle. Innov. Infrastruct. Solut..

[3] Siddika A., Amin M.R., Rayhan M.A., Islam M.S., Al Mamun M.A., Alyousef R., Amran Y.H.M. (2021). Performance of sustainable green concrete incorporated with fly ash rice husk ash and stone dust. Acta Polytech..

[4] Assaedi H., Alomayri T., Siddika A., Shaikh F., Alamri H., Subaer S., Low I.-M. (2019). Effect of Nanosilica on Mechanical Properties and Microstructure of PVA Fiber-Reinforced Geopolymer Composite (PVA-FRGC). Materials.

[5] Siddika A., Al Mamun M.A., Alyousef R., Mohammadhosseini H. (2021). State-of-the-art-review on rice husk ash: A supplementary cementitious material in concrete. J. King Saud Univ. Eng. Sci..

[6] Luhar S., Cheng T.-W., Nicolaides D., Luhar I., Panias D., Sakkas K. (2019). Valorisation of glass wastes for the development of geopolymer composites—Durability, thermal and microstructural properties: A review. Constr. Build. Mater..

[7] Burciaga-Díaz O., Durón-Sifuentes M., Díaz-Guillén J.A., Escalante-García J.I. (2020). Effect of waste glass incorporation on the properties of geopolymers formulated with low purity metakaolin. Cem. Concr. Compos..

[8] Siddika A., Al Mamun M.A., Ali M.H. (2018). Study on concrete with rice husk ash. Innov. Infrastruct. Solut..

[9] Siddika A., Al Mamun M.A., Ferdous W., Saha A.K., Alyousef R. (2020). 3D-printed concrete: Applications, performance, and challenges. J. Sustain. Cem. Mater..

[10] Trends in Solid Waste Management. https://datatopics.worldbank.org/what-a-waste/trends_in_solid_waste_management.html.

[11] Harder I.J. Glass Recycling—Current Market Trends—Recovery. https://www.recovery-worldwide.com/en/artikel/glass-recycling-current-market-trends_3248774.html.

[12] What a Waste: Australia Needs to Confront Recycling Crisis. https://www.news.com.au/technology/environment/australias-reliance-on-sending-waste-overseas-for-recycling-is-fuelling-a-crisis-in-the-industry/news-story/2921f34b88adcc7ad8925ca9430367ad.

[13] Hama S.M., Mahmoud A.S., Yassen M.M. (2019). Flexural behavior of reinforced concrete beam incorporating waste glass powder. Structures.

[14] Jiang Y., Ling T.-C., Mo K.H., Shi C. (2019). A critical review of waste glass powder—Multiple roles of utilization in cement-based materials and construction products. J. Environ. Manag..

[15] Heriyanto E., Pahlevani F., Sahajwalla V. (2018). From waste glass to building materials—An innovative sustainable solution for waste glass. J. Clean. Prod..

[16] Vafaei M., Allahverdi A. (2017). Durability of Geopolymer Mortar Based on Waste-Glass Powder and Calcium Aluminate Cement in Acid Solutions. J. Mater. Civ. Eng..

[17] Al-Akhras N.M. (2012). Performance of Glass Concrete Subjected to Freeze-Thaw Cycling. Open Constr. Build. Technol. J..

[18] Hajimohammadi A., Ngo T., Vongsvivut J. (2019). Interfacial chemistry of a fly ash geopolymer and aggregates. J. Clean. Prod..

[19] Jubeh A.I., Al Saffar D.M., Tayeh B.A. (2019). Effect of recycled glass powder on properties of cementitious materials contains styrene butadiene rubber. Arab. J. Geosci..

[20] Aliabdo A.A., Abd Elmoaty A.E.M., Aboshama A.Y. (2016). Utilization of waste glass powder in the production of cement and concrete. Constr. Build. Mater..

[21] Hajimohammadi A., Ngo T., Kashani A. (2018). Glass waste versus sand as aggregates: The characteristics of the evolving geopolymer binders. J. Clean. Prod..

[22] Topçu İ.B., Canbaz M. (2004). Properties of concrete containing waste glass. Cem. Concr. Res..

[23] Andiç-Çakır Ö., Üzüm O., Yüksel C., Sarikanat M. (2016). Waste glass aggregate for cementitious and polymer concrete. Proc. Inst. Civ. Eng. Constr. Mater..

[24] Williamson T., Juenger M.C.G. (2016). The role of activating solution concentration on alkali–silica reaction in alkali-activated fly ash concrete. Cem. Concr. Res..

[25] Hilton B., Bawden K., Winnebeck K., Chandrasiri C., Ariyachandra E., Peethamparan S. (2019). The functional and environmental performance of mixed cathode ray tubes and recycled glass as partial replacement for cement in concrete. Resour. Conserv. Recycl..

[26] Si R., Dai Q., Guo S., Wang J. (2020). Mechanical property, nanopore structure and drying shrinkage of metakaolin-based geopolymer with waste glass powder. J. Clean. Prod..

[27] Lee H., Hanif A., Usman M., Sim J., Oh H. (2018). Performance evaluation of concrete incorporating glass powder and glass sludge wastes as supplementary cementing material. J. Clean. Prod..

[28] Paul S.C., Šavija B., Babafemi A.J. (2018). A comprehensive review on mechanical and durability properties of cement-based materials containing waste recycled glass. J. Clean. Prod..

[29] Federico L.M., Chidiac S.E. (2009). Waste glass as a supplementary cementitious material in concrete—Critical review of treatment methods. Cem. Concr. Compos..

[30] Kazmi D., Williams D.J., Serati M. (2020). Waste glass in civil engineering applications—A review. Int. J. Appl. Ceram. Technol..

[31] Luhar S., Cheng T.-W., Nicolaides D., Luhar I., Panias D., Sakkas K. (2019). Valorisation of glass waste for development of Geopolymer composites—Mechanical properties and rheological characteristics: A review. Constr. Build. Mater..

[32] Ling T.-C., Poon C.-S. (2014). Use of recycled CRT funnel glass as fine aggregate in dry-mixed concrete paving blocks. J. Clean. Prod..

[33] Arabi N., Meftah H., Amara H., Kebaïli O., Berredjem L. (2019). Valorization of recycled materials in development of self-compacting concrete: Mixing recycled concrete aggregates—Windshield waste glass aggregates. Constr. Build. Mater..

[34] Liu G., Florea M.V.A., Brouwers H.J.H. (2019). Characterization and performance of high volume recycled waste glass and ground granulated blast furnace slag or fly ash blended mortars. J. Clean. Prod..

[35] Shi C., Wu Y., Riefler C., Wang H. (2005). Characteristics and pozzolanic reactivity of glass powders. Cem. Concr. Res..

[36] ASTM International (2003). ACTM C 618-03 Standard Specification for Coal Fly Ash and Raw or Calcined Natural Pozzolan for Use in Concrete.

[37] Ismail Z.Z., AL-Hashmi E.A. (2009). Recycling of waste glass as a partial replacement for fine aggregate in concrete. Waste Manag..

[38] Karamberi A., Moutsatsou A. (2005). Participation of coloured glass cullet in cementitious materials. Cem. Concr. Compos..

[39] Idir R., Cyr M., Tagnit-Hamou A. (2010). Use of fine glass as ASR inhibitor in glass aggregate mortars. Constr. Build. Mater..

[40] Shao Y., Lefort T., Moras S., Rodriguez D. (2000). Studies on concrete containing ground waste glass. Cem. Concr. Res..

[41] Du H., Tan K.H. (2017). Properties of high volume glass powder concrete. Cem. Concr. Compos..

[42] He Z., Zhan P., Du S., Liu B., Yuan W. (2019). Creep behavior of concrete containing glass powder. Compos. Part B Eng..

[43] Islam G.M.S., Rahman M.H., Kazi N. (2017). Waste glass powder as partial replacement of cement for sustainable concrete practice. Int. J. Sustain. Built Environ..

[44] Mirzahosseini M., Riding K.A. (2014). Effect of curing temperature and glass type on the pozzolanic reactivity of glass powder. Cem. Concr. Res..

[45] Hajimohammadi A., Ngo T., Kashani A. (2018). Sustainable one-part geopolymer foams with glass fines versus sand as aggregates. Constr. Build. Mater..

[46] Toniolo N., Taveri G., Hurle K., Roether J.A., Ercole P., Dlouhý I., Boccaccini A.R. (2017). Fly-ash-based geopolymers: How the addition of recycled glass or red mud waste influences the structural and mechanical properties. J. Ceram. Sci. Technol..

[47] El-Naggar M.R., El-Dessouky M.I. (2017). Re-use of waste glass in improving properties of metakaolin-based geopolymers: Mechanical and microstructure examinations. Constr. Build. Mater..

[48] Novais R.M., Ascensão G., Seabra M.P., Labrincha J.A. (2016). Waste glass from end-of-life fluorescent lamps as raw material in geopolymers. Waste Manag..

[49] Torres-Carrasco M., Puertas F. (2015). Waste glass in the geopolymer preparation. Mechanical and microstructural characterisation. J. Clean. Prod..

[50] Khale D., Chaudhary R. (2007). Mechanism of geopolymerization and factors influencing its development: A review. J. Mater. Sci..

[51] Menchaca-Ballinas L.E., Escalante-Garcia J.I. (2019). Low CO_2_ emission cements of waste glass activated by CaO and NaOH. J. Clean. Prod..

[52] Cyr M., Idir R., Poinot T. (2012). Properties of inorganic polymer (geopolymer) mortars made of glass cullet. J. Mater. Sci..

[53] Torres-Carrasco M., Puertas F. (2017). Waste glass as a precursor in alkaline activation: Chemical process and hydration products. Constr. Build. Mater..

[54] Liu Y., Shi C., Zhang Z., Li N. (2019). An overview on the reuse of waste glasses in alkali-activated materials. Resour. Conserv. Recycl..

[55] Vafaei M., Allahverdi A. (2017). High strength geopolymer binder based on waste-glass powder. Adv. Powder Technol..

[56] Zhang S., Keulen A., Arbi K., Ye G. (2017). Waste glass as partial mineral precursor in alkali-activated slag/fly ash system. Cem. Concr. Res..

[57] Wang H.Y., Huang W.L. (2010). A study on the properties of fresh self-consolidating glass concrete (SCGC). Constr. Build. Mater..

[58] Lu J., Duan Z., Poon C.S. (2017). Combined use of waste glass powder and cullet in architectural mortar. Cem. Concr. Compos..

[59] Lu J.-X., Zheng H., Yang S., He P., Poon C.S. (2019). Co-utilization of waste glass cullet and glass powder in precast concrete products. Constr. Build. Mater..

[60] Kashani A., Ngo T.D., Hajimohammadi A. (2019). Effect of recycled glass fines on mechanical and durability properties of concrete foam in comparison with traditional cementitious fines. Cem. Concr. Compos..

[61] Omran A., Tagnit-Hamou A. (2016). Performance of glass-powder concrete in field applications. Constr. Build. Mater..

[62] Wang Z., Shi C., Song J. (2009). Effect of glass powder on chloride ion transport and alkali-aggregate reaction expansion of lightweight aggregate concrete. J. Wuhan Univ. Technol. Sci. Ed..

[63] Thomas M.D.A., Hooton R.D., Scott A., Zibara H. (2012). The effect of supplementary cementitious materials on chloride binding in hardened cement paste. Cem. Concr. Res..

[64] Tayeh B.A., Al Saffar D.M., Aadi A.S., Almeshal I. (2019). Sulphate resistance of cement mortar contains glass powder. J. King Saud Univ. Eng. Sci..

[65] Mostofinejad D., Hosseini S.M., Nosouhian F., Ozbakkaloglu T., Tehrani B.N. (2020). Durability of concrete containing recycled concrete coarse and fine aggregates and milled waste glass in magnesium sulfate environment. J. Build. Eng..

[66] Sales R.B.C., Sales F.A., Figueiredo E.P., dos Santos W.J., Mohallem N.D.S., Aguilar M.T.P. (2017). Durability of Mortar Made with Fine Glass Powdered Particles. Adv. Mater. Sci. Eng..

[67] Özkan Ö., Yüksel İ. (2008). Studies on mortars containing waste bottle glass and industrial by-products. Constr. Build. Mater..

[68] Torres-Carrasco M., Tognonvi M.T., Tagnit-Hamou A., Puertas F. (2015). Durability of Alkali-Activated Slag Concretes Prepared Using Waste Glass as Alternative Activator. ACI Mater. J..

[69] Wang C.-C., Wang H.-Y., Chen B.-T., Peng Y.-C. (2017). Study on the engineering properties and prediction models of an alkali-activated mortar material containing recycled waste glass. Constr. Build. Mater..

[70] Vafaei M., Allahverdi A., Dong P., Bassim N. (2018). Acid attack on geopolymer cement mortar based on waste-glass powder and calcium aluminate cement at mild concentration. Constr. Build. Mater..

[71] Zhang Z., Provis J.L., Reid A., Wang H. (2014). Fly ash-based geopolymers: The relationship between composition, pore structure and efflorescence. Cem. Concr. Res..

[72] Bobirică C., Shim J.-H., Park J.-Y. (2018). Leaching behavior of fly ash-waste glass and fly ash-slag-waste glass-based geopolymers. Ceram. Int..

[73] Wang W.-C., Chen B.-T., Wang H.-Y., Chou H.-C. (2016). A study of the engineering properties of alkali-activated waste glass material (AAWGM). Constr. Build. Mater..

[74] Abendeh R., Baker M.B., Salem Z.A., Ahmad H. (2015). The Feasibility of Using Milled Glass Wastes in Concrete to Resist Freezing-Thawing Action. Int. J. Civ. Environ. Eng..

[75] Siddika A., Al Mamun M.A., Alyousef R., Amran Y.H.M., Aslani F., Alabduljabbar H. (2019). Properties and utilizations of waste tire rubber in concrete: A review. Constr. Build. Mater..

[76] Carsana M., Frassoni M., Bertolini L. (2014). Comparison of ground waste glass with other supplementary cementitious materials. Cem. Concr. Compos..

[77] Schwarz N., DuBois M., Neithalath N. (2007). Electrical conductivity based characterization of plain and coarse glass powder modified cement pastes. Cem. Concr. Compos..

[78] Cai J., Pan J., Li X., Tan J., Li J. (2020). Electrical resistivity of fly ash and metakaolin based geopolymers. Constr. Build. Mater..

[79] Tan K.H., Du H. (2013). Use of waste glass as sand in mortar: Part I—Fresh, mechanical and durability properties. Cem. Concr. Compos..

[80] Cassar J., Camilleri J. (2012). Utilisation of imploded glass in structural concrete. Constr. Build. Mater..

[81] Kou S.C., Poon C.S. (2009). Properties of self-compacting concrete prepared with recycled glass aggregate. Cem. Concr. Compos..

[82] Kim K.-H., Jeon S.-E., Kim J.-K., Yang S. (2003). An experimental study on thermal conductivity of concrete. Cem. Concr. Res..

[83] Poutos K.H., Alani A.M., Walden P.J., Sangha C.M. (2008). Relative temperature changes within concrete made with recycled glass aggregate. Constr. Build. Mater..

[84] Abdollahnejad Z., Dalvand A., Mastali M., Luukkonen T., Illikainen M. (2019). Effects of waste ground glass and lime on the crystallinity and strength of geopolymers. Mag. Concr. Res..

[85] Tchakouté H.K., Rüscher C.H., Kong S., Kamseu E., Leonelli C. (2017). Thermal Behavior of Metakaolin-Based Geopolymer Cements Using Sodium Waterglass from Rice Husk Ash and Waste Glass as Alternative Activators. Waste Biomass Valoriz..

[86] Temuujin J., Minjigmaa A., Rickard W., van Riessen A. (2012). Thermal properties of spray-coated geopolymer-type compositions. J. Therm. Anal. Calorim..

[87] Bai C., Li H., Bernardo E., Colombo P. (2019). Waste-to-resource preparation of glass-containing foams from geopolymers. Ceram. Int..

[88] Pan Z., Tao Z., Murphy T., Wuhrer R. (2017). High temperature performance of mortars containing fine glass powders. J. Clean. Prod..

[89] Fernandes H.R., Tulyaganov D.U., Ferreira J.M.F. (2009). Preparation and characterization of foams from sheet glass and fly ash using carbonates as foaming agents. Ceram. Int..

[90] Owoeye S.S., Matthew G.O., Ovienmhanda F.O., Tunmilayo S.O. (2020). Preparation and characterization of foam glass from waste container glasses and water glass for application in thermal insulations. Ceram. Int..

[91] Patel D., Shrivastava R., Tiwari R.P., Yadav R.K. (2020). Properties of cement mortar in substitution with waste fine glass powder and environmental impact study. J. Build. Eng..

[92] Bignozzi M.C., Saccani A., Barbieri L., Lancellotti I. (2015). Glass waste as supplementary cementing materials: The effects of glass chemical composition. Cem. Concr. Compos..

[93] Zhu H., Chen W., Zhou W., Byars E.A. (2009). Expansion behaviour of glass aggregates in different testing for alkali-silica reactivity. Mater. Struct..

[94] Lee G., Ling T.C., Wong Y.L., Poon C.S. (2011). Effects of crushed glass cullet sizes, casting methods and pozzolanic materials on ASR of concrete blocks. Constr. Build. Mater..

[95] Corinaldesi V., Gnappi G., Moriconi G., Montenero A. (2005). Reuse of ground waste glass as aggregate for mortars. Waste Manag..

[96] Tamanna N., Tuladhar R., Sivakugan N. (2020). Performance of recycled waste glass sand as partial replacement of sand in concrete. Constr. Build. Mater..

[97] Taha B., Nounu G. (2009). Utilizing Waste Recycled Glass as Sand/Cement Replacement in Concrete. J. Mater. Civ. Eng..

[98] Kawamura M., Fuwa H. (2003). Effects of lithium salts on ASR gel composition and expansion of mortars. Cem. Concr. Res..

[99] Lee J.-C., Jang B.-K., Shon C.-S., Kim J.-H., Chung C.-W. (2019). Potential use of borosilicate glass to make neutron shielding mortar: Enhancement of thermal neutron shielding and strength development and mitigation of alkali-silica reaction. J. Clean. Prod..

[100] Du H., Tan K.H. (2014). Effect of particle size on alkali–silica reaction in recycled glass mortars. Constr. Build. Mater..

[101] Mehta A., Ashish D.K. (2020). Silica fume and waste glass in cement concrete production: A review. J. Build. Eng..

[102] Olofinnade O.M., Ede A.N., Ndambuki J.M., Ngene B.U., Akinwumi I.I., Ofuyatan O. (2018). Strength and microstructure of eco-concrete produced using waste glass as partial and complete replacement for sand. Cogent Eng..

[103] Lu J.-X., Poon C.S. (2019). Recycling of waste glass in construction materials. New Trends in Eco-Efficient and Recycled Concrete.

