# Correction: Siddika et al. Waste Glass in Cement and Geopolymer Concretes: A Review on Durability and Challenges. *Polymers* 2021, *13*, 2071

**DOI:** 10.3390/polym14061068

**Published:** 2022-03-08

**Authors:** Ayesha Siddika, Ailar Hajimohammadi, Md. Abdullah Al Mamun, Rayed Alyousef, Wahid Ferdous

**Affiliations:** 1 School of Civil and Environmental Engineering, University of New South Wales (UNSW Sydney), Sydney, NSW 2052, Australia; a.siddika@unsw.edu.au; 2 Department of Civil Engineering, Rajshahi University of Engineering & Technology, Rajshahi 6204, Bangladesh; mamun_05ce7@yahoo.com; 3 Department of Civil Engineering, College of Engineering, Prince Sattam Bin Abdulaziz University, Alkharj 11942, Saudi Arabia; r.alyousef@psau.edu.sa; 4 Centre for Future Materials, University of Southern Queensland, Toowoomba, QLD 4350, Australia

The authors wish to make the following corrections to this paper [1]: in the original version of our article, the unit of production rate of glass and waste glass was mistakenly written in “metric tons” but should be corrected to “million tons”. The corrected sentences are presented below:

**In Abstract:** Every year, the world is producing around 100 million tons of waste glass (WG), the majority of them are going to landfills that create massive environmental problems.

**In Introduction:** (first sentence of second paragraph)

Globally, around 130 million tons of glass are being produced each year among which approximately 100 million tons are being discarded as waste. 

The authors apologize for this error and state that the scientific conclusions are unaffected. The original article has been updated.
